# Expanded access to viral load testing and use of second line regimens in Haiti: time trends from 2010–2017

**DOI:** 10.1186/s12879-020-04978-9

**Published:** 2020-04-16

**Authors:** Yu Wang, Scott Barnhart, Kesner Francois, Ermane Robin, Mireille Kalou, Georges Perrin, Lara Hall, Jean Baptiste Koama, Elisma Marinho, Jean Gabriel Balan, Jean Guy Honoré, Nancy Puttkammer

**Affiliations:** 1 Department of Global Health, University of Washington, Seattle, USA; 2 Departments of Medicine and Global Health, University of Washington, Seattle, USA; 3 National AIDS Control Program, Haiti Ministry of Public Health and Population (PNLS/MSPP), Port-au-Prince, Haiti; 4 Division of Global HIV and Tuberculosis Haiti, US Centers for Disease Control and Prevention, Atlanta, USA; 5 Centre Haïtien pour le Renforcement du Système de Santé, Port-au-Prince, Haiti

**Keywords:** Viral load, VL, Second-line regimen, ART, Antiretroviral therapy, ART adherence, Haiti, HIV

## Abstract

**Background:**

Haiti initiated the scale-up of HIV viral load (VL) testing in 2015–2016, with plans to achieve 100% coverage for all patients on antiretroviral therapy (ART) for treatment of HIV/AIDS. In the absence of HIV drug susceptibility testing, VL testing is a key tool for monitoring response to ART and optimizing treatment results. This study describes trends in expanded use of VL testing, VL results, and use of second-line ART regimens, and explores the association between VL testing and second-line regimen switching in Haiti from 2010 to 2017.

**Methods:**

We conducted a retrospective cohort study with 66,042 patients drawn from 88 of Haiti’s 160 national ART clinics. Longitudinal data from the iSanté electronic data system was used to analyze the trends of interest. We described patients’ VL testing status in five categories based on up to two most recent VL test results: no test; suppressed; unsuppressed followed by no test; re-suppressed; and confirmed failure. Among those with confirmed failure, we described ART adherence level. Finally, we used Cox proportional hazards regression to estimate the risk of second-line regimen switching by VL testing status, after adjusting for other individual characteristics.

**Results:**

The number of patients who had tests done increased annually from 11 in 2010 to 18,828 in the first 9 months of 2017, while the number of second-line regimen switches rose from 21 to 279 during this same period. Compared with patients with no VL test, the hazard ratio (HR) for switching to a second-line regimen was 22.2 for patients with confirmed VL failure (95% confidence interval [CI] for HR: 18.8–26.3; *p* < 0.005) after adjustment for individual characteristics. Among patients with confirmed VL failure, 44.7% had strong adherence, and fewer than 20% of patients switched to a second-line regimen within 365 days of VL failure.

**Conclusions:**

Haiti has significantly expanded access to VL testing since 2016. In order to promote optimal patient health outcomes, it is essential for Haiti to continue broadening access to confirmatory VL testing, to expand evidence-based initiatives to promote strong ART adherence, and to embrace timely switching for patients with confirmed ART failure despite strong ART adherence.

## Background

HIV/AIDS is a life-threatening disease which, if untreated, destroys the immune system, leaving those infected susceptible to opportunistic infection and early death. Antiretroviral therapy (ART) for treatment of HIV, when used consistently, suppresses HIV replication and prevents progression of HIV disease [1], leading to highly successful clinical, immunologic, and virologic outcomes for patients with HIV/AIDS. The scale-up and appropriate management of patients on ART is essential for individual and population health [2, 3]. The Joint United Nations Programme on HIV/AIDS and partners launched the 95–95–95 targets for HIV epidemic control, with the aim that by 2030: 95% of all people living with HIV know their HIV status, 95% of all people diagnosed with HIV receive sustained ART, and 95% of all people on ART achieve viral suppression [4].

The initial treatment for most HIV patients is a first-line ART regimen, but either weak adherence or the presence of drug resistance can cause virologic failure. The number of patients who experience virologic failure and who need second-line therapy has increased [5–7]. Second-line treatments can be used to treat resistant forms of HIV successfully, but these regimens are more expensive than first-line regimens. Besides cost considerations, first-line regimens tend to be familiar to clinicians, to have favorable side effect profiles, to have broad applicability, and to be available in fixed-dose combinations with lower pill burden for patients. Therefore, there is a strong desire in resource-limited settings to optimize outcomes of first-line regimens so that the use of second- and third-line regimens is limited [8]. However, for patients with resistant forms of HIV, switching to second-line regimens is appropriate, and delays in switching can result in prolonged viremia, which leads to morbidity and mortality, as well as onward transmission of HIV.

To maximize the duration of first-line treatment and to justify switching to a second-line regimen when drug resistance is suspected, ART monitoring is necessary. In the past, in resource-limited settings, clinician decisions about ART regimen switching tended to rely on the World Health Organization (WHO) clinical and immunological criteria (CD4 monitoring) for ART failure. However, these criteria suffer from poor sensitivity and specificity in detecting true cases of treatment failure, leading to delayed detection of failure and low rates of switching to second-line ART regimens. Routine viral load (VL) testing is the preferred modality for ART monitoring and has been demonstrated to improve health outcomes of HIV patients through timely detection of treatment failure [9–12]. It has long been a standard of care in wealthy countries [13] and is now recommended by WHO for routine use in low and middle-income countries [14]. It must be noted that VL testing is not able to differentiate between failure due to poor adherence or to drug resistance, but in the absence of widely-available drug susceptibility and resistance testing in many resource-limited settings, VL testing is an essential tool in HIV care.

After sub-Saharan Africa, the Caribbean region has the second highest HIV prevalence globally [15], and Haiti has the greatest burden of HIV infection within the Caribbean region. In 1993, the prevalence of HIV among adults aged 15–49 in Haiti was approximately 2.6% for males and 2.4% for females and ART was not yet widely available [16]. Since then, substantial progress has been made in all aspects of the HIV care continuum, including HIV testing, linkage to care, and treatment. HIV prevalence decreased to 2.3% among adult women and 1.5% among adult men by 2017 [17, 18]. In Haiti, a total of 92,409 patients were receiving ART in 2017, representing over 62% of the estimated 149,047 people living with HIV in the country [19].

With support by the US President’s Emergency Plan for AIDS Relief (PEPFAR) and other funding sources, Haiti and other resource-limited countries have taken steps to expand access to VL testing. In 2015–16, Haiti’s Ministry of Public Health and Population (MSPP) initiated the scale-up of VL testing for all ART patients after 6 months on treatment and annually thereafter. In July 2016, Haiti adopted the “Test and Start” approach to ART initiation, making all patients immediately eligible for ART and thereby broadening the population of patients requiring regular VL testing [20]. In 2018–19, Haiti sought to achieve 100% VL coverage for all patients in the national ART program [19].

Monitoring expanded access to VL testing and examining whether this has improved evidence-based clinical management, including timely switching to second-line regimens, are important goals of policy evaluation in Haiti. The objectives of the present research study are: 1) to describe time trends in VL tests and results; 2) to describe time trends in switching from first- to second-line regimens; and 3) to explore the association between VL testing and second-line regimen switching from 2010 to 2017 in Haiti. This time frame followed Haiti’s devastating 2010 earthquake, a period when HIV clinical guidelines and resources for virologic monitoring of ART evolved toward routine VL testing.

## Methods

### Design and settings

This is a retrospective cohort study using longitudinal electronic medical record (EMR) data from the iSanté data system. iSanté is the largest of three EMRs in Haiti, which contains records for approximately 70% of ART patients in Haiti [21, 22]. Patient data is captured in the local ART sites and then sent to a central data repository. Data on the study cohort was drawn from 88 out of 126 clinics using the iSanté EMR out of 162 national ART sites, as of October 2017 [23].

### Participants

ART sites were included in our study based on the timeliness of data uploaded to the central iSanté data repository. Health facilities with fewer than 80% of patient visit forms saved to the iSanté central server within 90 days of the patient’s visit were excluded from the analysis. The goal of excluding these sites was to ensure that the data reflected the reality of care processes taking place at the clinics, thereby limiting bias when analyzing the total number of VL tests, number and percentage of patients with at least one VL test, as well as outcome of the VL tests. Three prison clinics were also excluded from the analysis, based on restrictions to secondary data use from these sites. Eligible patients were adults aged 16 and above who initiated a standard first-line ART regimen between January 1, 2010 and September 30, 2017 and who completed at least 6 months on ART. The study excluded patients who initiated ART on a second-line regimen. In each calendar year, active patients were those who picked up ART medication at least once that year. Patients were divided into annual ART cohorts according to their year of ART initiation.

### Viral load testing

Haiti national ART guidelines recommend VL testing after 6 months on ART and annually thereafter. Haiti uses the “Generic HIV Charge Virale” viral load assay (Biocentric, Bandol France) to measure plasma VL, with a lower limit of detection of 416 copies/ml when 250uL of plasma is used for testing; however, only values of ≥1000 copies/ml are considered to be unsuppressed per Haiti’s national guidelines [24]. ART patients are recommended to continue on a first-line regimen with a suppressed VL result. If they receive an unsuppressed VL result, patients are expected to receive enhanced adherence counseling with psychologists, social workers, nurses, pharmacists, or community health workers and then to receive a repeat VL test three to 6 months later. If the result confirms ART failure, the patients are eligible for switching to second-line regimens.

We classified VL status into five categories based upon the most recent VL test result for every patient and, if available, the test result immediately preceding it. The five categories were: 1) no test; 2) suppressed (VL test result < 1000 copies/ml with no prior test or with a prior suppressed result); 3) unsuppressed (VL result > 1000 copies/ml with no prior test or with a prior suppressed result); 4) re-suppressed (VL test result < 1000 copies/ml following an unsuppressed result); and 5) confirmed failure (VL result > 1000 copies/ml following a previous unsuppressed result).

### ART regimens

The preferred first-line ARV regimens in Haiti since 2008 have included two nucleoside reverse transcriptase inhibitors (NRTIs) and one non-nucleoside reverse transcriptase inhibitor (NNRTI). Prior to 2013, the preferred first-line regimens consisted of tenofovir plus emtricitabine plus efavirenz or nevirapine. After 2013, the preferred first-line regimens consisted of tenofovir plus lamivudine plus efavirenz. Several alternative first line regimens were recognized for pediatric patients of less than 10 years, patients with renal insufficiency, or patients with adverse drug reactions. Second-line regimens were defined as two NRTIs combined with a ritonavir-boosted protease inhibitor [25]. Prior to 2013 the main second-line regimen contained tenofovir plus emtricitabine plus ritonavir-boosted lopinavir. After 2013, the main second-line regimens contained tenofovir plus lamivudine plus ritonavir-boosted lopinavir or ritonavir-boosted atazanavir [26–28]. The 2017 national ART guidelines expanded ART eligibility to all PLWH and added several second-line regimen options, but did not change the preferred first and second-line ART regimens.

For this study, we defined ART regimens as first or second-line according to the national ART guidelines in place when the medication was picked up: the 2008 national ART guideline covered ART medications picked up from January 2010 to December 2012; the 2013 guideline covered medications picked up from January 2013 to December 2016; and the 2017 guideline covered medications picked up in 2017.

### Adherence

Following an unsuppressed VL result, clinicians are expected to encourage improved adherence and to use standardized documentation of adherence in patients with confirmed ART failure to justify a regimen switch. In this study, we assessed adherence in the 180 days prior to confirmed cases of VL failure using the medication possession ratio (MPR), or the percentage of days that patients had medication in their possession. If the MPR was greater than 90%, the patients were considered as having strong adherence, and if the MPR was less than 90%, the patients were considered as having weak adherence [29–34].

### Data analysis

#### Time trends in viral load testing and results

The total number of VL tests, the number and proportion of unsuppressed tests, the number of total patients in care, and the number and proportion of patients who had at least one VL test are described using frequency tables. We used these results to present the VL cascade by calendar year, to illuminate time trends in VL testing and results. We also analyzed VL testing and virologic outcomes by ART cohort for the 2010 to 2017 cohorts. In these ART cohort analyses, we first used the Kaplan-Meier method to assess time from ART initiation to first VL test by ART cohort, among all ART patients. Next, we considered the sub-set of patients with at least 18 months of possible follow-up time after ART initiation, since VL testing was not always consistently administered 6 months after ART initiation as directed by national guidelines. We characterized the VL status of the 7 ART cohorts (cohort 2010 to cohort 2016) using the results of up to two most recent tests available for each person.

#### Time trends in second-line regimen switching

We described the rate of switching to second-line regimens by ART cohort, first among all patients and then among those observed to be on ART for at least 18 months, for the 2010 to 2016 ART cohorts. We explored the VL status among switching patients. Finally, we used the Kaplan-Meier method to estimate the proportion of patients switching to second-line regimens by two time-to-event outcomes: time from initial unsuppressed VL test to second-line switching, as well as time from confirmed failure to second-line switching.

#### Association between VL status and second-line regimen switching

We used a Cox regression model to assess the relationship between VL status (using the five categories described above) and second-line regimen switching among the 2010 to 2017 ART cohorts. In this analysis, the VL status was a time-varying variable. We explored the association after adjusting for age, gender, WHO stage at ART initiation, and year of ART initiation. There were no missing values for year of ART initiation or age (since we excluded cases with missing age during the data exclusion process). We created a missing indicator category for cases with missing gender or WHO stage. For this analysis, we considered all available patients and all available VL tests prior to switching or administrative censoring (whichever came first), rather than limiting the analysis to patients with at least 18 months of follow up and limiting to the two most recent VL tests. Thus, patients had differing durations of follow up.

When analyzing the outcomes of VL testing, time taken from ART start to initial VL test, and time from initial unsuppressed VL test to second-line regimen switching (Tables 2, 3 and 4), we used a cohort method to report the results, in order to show the differences among patients by ART start year.

## Results

**Fig. 1:**
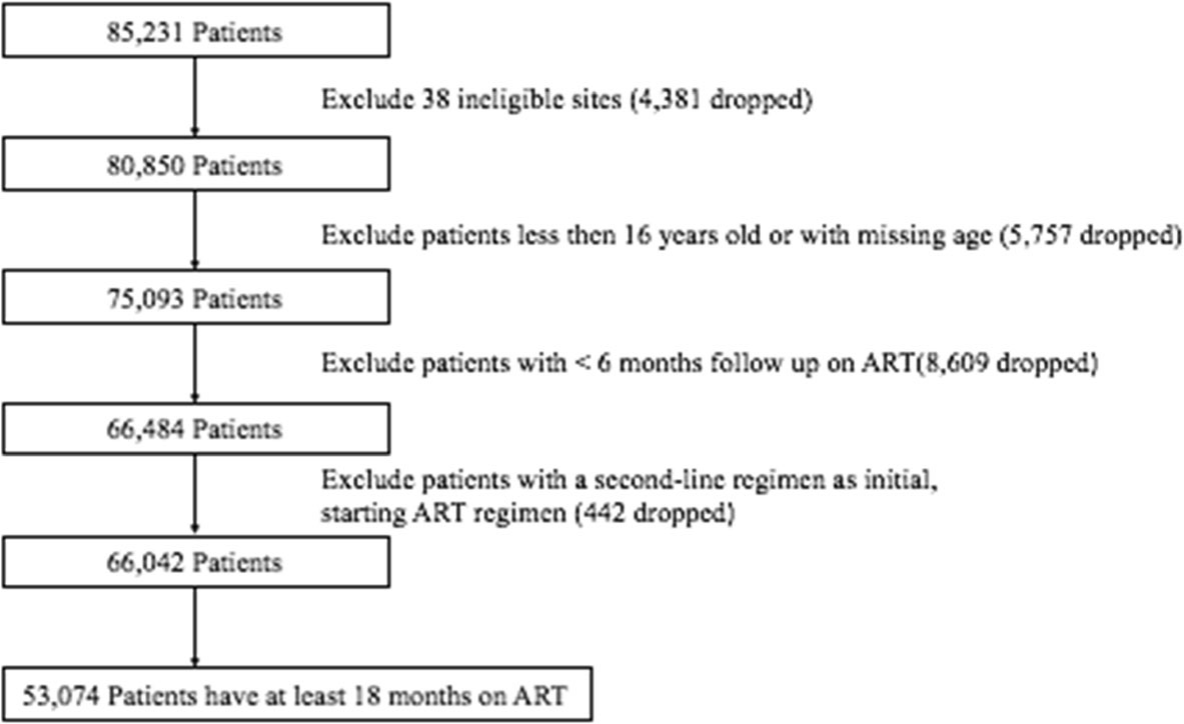
Patient Inclusion

Out of 126 sites with data recorded in the iSanté system, there were 88 eligible sites with 66,042 patients from the 2010 to 2017 ART cohorts (Fig. 1). The median age at ART initiation was 36.0 years (interquartile range [IQR]: 18.0-45.1 years), 64.2% were female, and 21.4 and 32.9% were WHO stage III and stage IV respectively. Among the patients in the study, 53,074 from the 2010 to 2016 ART cohorts had at least 18 months of observed follow-up time. Patient characteristics are shown in Table 1.

**Table 1:** Patient Characteristics

Categories		Total ART patients	Patients with at least 18 months on ART
Total		66,042	53,074
Age	16–24	8173 (12.4%)	6488 (12.2%)
	25–34	22,335 (33.8%)	17,858 (33.7%)
	35–44	18,828 (28.5%)	15,093 (28.4%)
	45–54	10,778 (16.3%)	8745 (16.5%)
	> 55	5928 (9.0%)	4890 (9.2%)
Gender	Female	42,603 (64.5%)	34,278 (64.6%)
	Male	23,409 (35.4%)	18,779 (35.4%)
	Missing	30 (0.1%)	17 (0.03%)
Year of ART initiation	2010	3914 (5.9%)	3914 (7.4%)
	2011	5863 (8.9%)	5863 (11.0%)
	2012	8641 (13.1%)	8641 (16.3%)
	2013	10,812 (16.3%)	10,812 (20.4%)
	2014	11,629 (17.6%)	11,629 (21.9%)
	2015	10,483 (15.9%)	10,325 (19.5%)
	2016	12,667 (19.2%)	1890 (3.5%)
	2017	2033 (3.1%)	n/a
WHO stage at ART initiation	Stage 1	12,879 (19.5%)	8346 (15.7%)
	Stage 2	10,983 (16.7%)	7818 (14.7%)
	Stage 3	14,217 (21.5%)	12,142 (22.9%)
	Stage 4	21,814 (33.0%)	20,137 (37.9%)
	Missing	6149 (9.3%)	4631 (8.8%)

### VL Cascade by calendar year

**Fig. 2:**
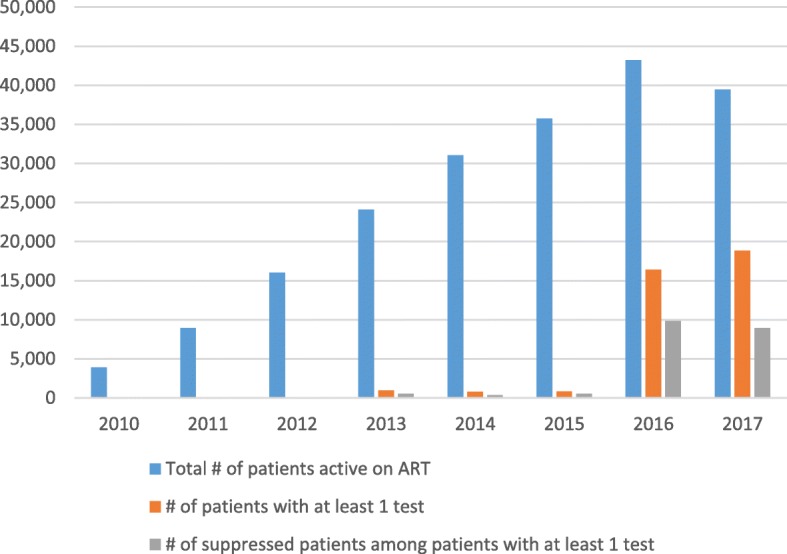
Viral Load cascade among active ART patients from 2010 to 2017

The total number of VL tests recorded in iSanté increased from 11 tests in 2010 to 20,221 in the first three quarters of 2017, while the number of patients with a VL test grew from 11 patients to 18,828 during during this time frame (Table 2). This represented expansion in the proportion of active patients with at least one VL test from 0.3% in 2010 to nearly half (47.7%) in 2017 (Fig. 2). The proportion of total VL tests with unsuppressed results fluctuated, with rates of 39.8 and 53.4% in 2016 and 2017 respectively, when the majority of VL tests were conducted (Table 2). The evolution of the VL cascade, showing the number and proportion of active patients with at least one test and the with suppressed results by calendar year, is shown in Fig. 2.

**Table 2:** Use of VL Testing by Calendar Year from 2010 to 2017 (*n* = 66,042 patients) ^a^

	2010	2011	2012	2013	2014	2015	2016	2017
Total # of tests	11	9	84	970	826	864	17,549	20,221
# and % of unsuppressed tests	7 (63.6%)	4 (44.4%)	15 (17.9%)	408 (42.1%)	428 (51.8%)	306 (35.4%)	6988 (39.8%)	10,790 (53.4%)
Total # of patients active on ART	3913	8943	16,003	24,099	31,078	35,772	43,229	39,469
# and % of patients with at least 1 test	11 (0.3%)	9 (0.1%)	64 (0.4%)	951 (3.9%)	786 (2.5%)	839 (2.3%)	16,392 (37.9%)	18,828 (47.7%)
# and % of suppressed patients among patients with at least 1 test	4 (36.4%)	5 (55.6%)	51 (79.7%)	547 (57.5%)	378 (48.1%)	544 (64.8%)	9864 (60.2%)	8968 (47.6%)

^a^Year 2017 includes data from January –September (9 months) only. Proportion shown of patients with at least one test is among active ART patients during the given calendar year

**Fig. 3:**
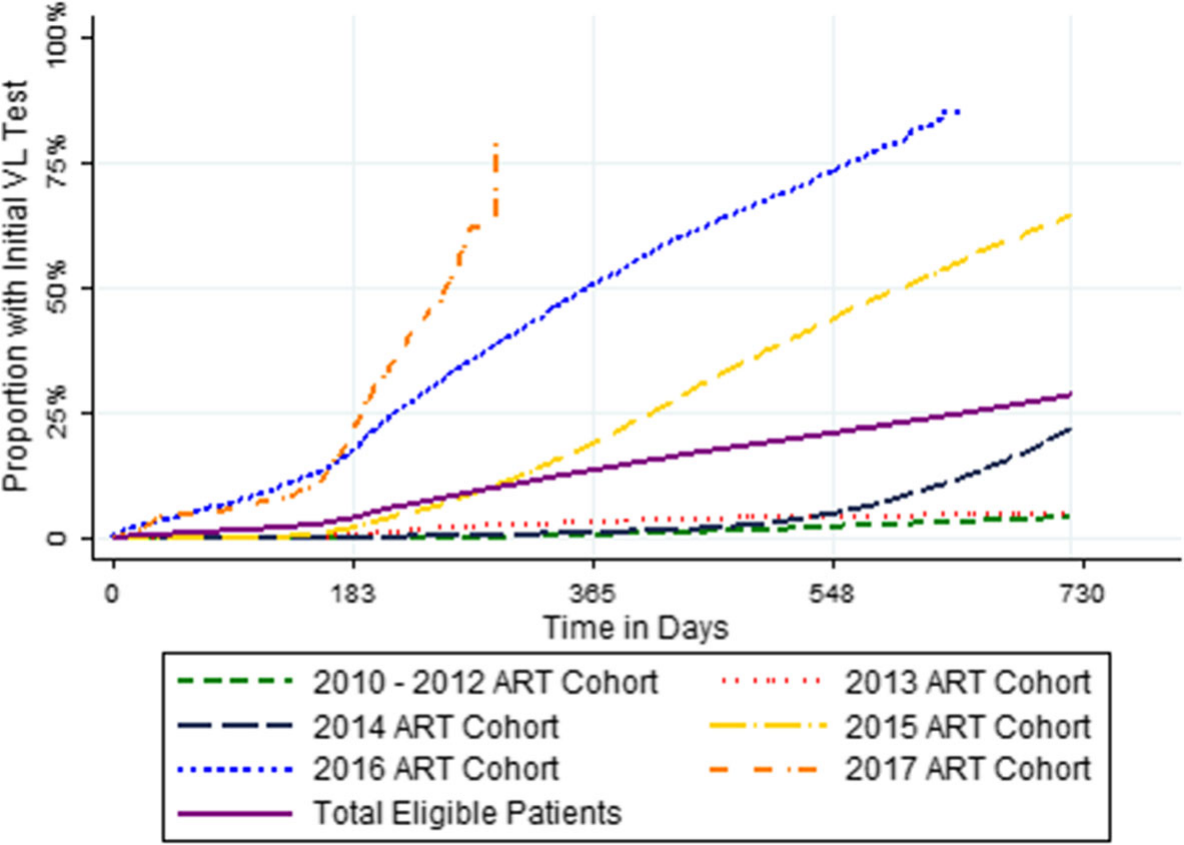
Kaplan-Meier Estimates of Time to Initial Viral Load Test (*n* = 66,042)

Figure 3 shows the timeliness of uptake of first VL test, by ART cohort. The cumulative probability of a first VL test was 28.5% after 2 years on ART (95% confidence interval [CI]: 28.1–28.9%). Patients in later ART cohorts were more likely to have a VL test within 6 months of ART initiation. For those with multiple tests, the median time between the two most recent tests was 336 days (IQR: 193–404 days) (result not shown).

### Viral load status by ART cohort

When considering the 53,074 patients with at least 18 months of follow-up across the seven annual ART cohorts from 2010 to 2016, 55.3% of patients had no test; 23.1% had a single test, and 15.6% had two or more tests (overall range: 0–7 tests; IQR: 0–2 tests). A total of 14.1% of patients experienced an unsuppressed result in one or both of two most recent VL tests (Table 3). Among those with any unsuppressed result, 59.5% had no follow-up confirmatory test after the unsuppressed result, 12.4% had a re-suppressed result, and 28.1% had a confirmed VL failure. For patients with confirmed VL failure, nearly half (44.7%) had strong ART adherence in the 180 days prior to the confirmed failure.

**Table 3:** Outcome of VL Test by ART Cohort Year (*n* = 53,074 patients)^a^

	2010 ART Cohort	2011 ART Cohort	2012 ART Cohort	2013 ART Cohort	2014 ART Cohort	2015 ART Cohort	2016 ART Cohort	Total
Total # of patients	3914	5863	8641	10,812	11,629	10,325	1890	53,074
% with no test	56.6%	55.6%	57.5%	58.0%	54.0%	52.7%	48.3%	55.3%
% suppressed	29.4%	30.1%	28.4%	28.2%	32.4%	32.8%	36.3%	30.6%
% unsuppressed^a^	14.0%	14.3%	14.1%	13.8%	13.6%	14.5%	15.4%	14.1%
Among patients with initial unsuppressed result								
# unsuppressed	549	841	1213	1494	1588	1492	291	7468
% re-suppressed	16.4%	12.0%	13.9%	12.6%	11.6%	10.9%	10.3%	12.4%
% unsuppressed followed by no test	50.6%	55.8%	57.3%	57.5%	61.2%	64.6%	70.1%	59.5%
% confirmed failure	33.0%	32.2%	28.8%	29.9%	27.2%	24.5%	19.6%	28.1%
Among patients with confirmed failure								
# confirmed failure	181	271	349	447	431	366	57	2102
% with strong adherence	47.0%	46.1%	47.9%	43.8%	43.9%	40.7%	49.1%	44.7%
% with weak adherence	53.0%	53.9%	52.1%	56.2%	56.1%	59.3%	50.9%	55.3%

^a^ Includes ART patients with at least 18 months of follow-up time (see Table 1 for characteristics of 53,074 ART patients). VL status is based on up to two most recent tests, and the unsuppressed group includes patients who had a single test with unsuppressed result, as well as patients who had two tests with an unsuppressed result on one or both tests

Patients who initiated ART earlier had a higher “no test” proportion. For the 2010 ART cohort, 56.6% of patients never had a VL test (2214 out of 3914), and this decreased to 48.3% for 2016 ART cohort (913 out of 1890). The proportion of patients with no confirmatory testing following an initial unsuppressed result was higher among later ART cohorts. For the 2010 ART cohort, 50.6% of unsuppressed patients had no confirmatory test (278 out of 549), and this number increased to 70.1% for 2016 ART cohort (204 out of 291) (Table 3).

### Switching to second-line ART

With the scale up of ART in Haiti, the absolute number of patients switching to second-line regimens increased with time. Among the 66,042 patients total, 1736 patients switched to a second-line regimen during 125,645 person-years (1.4 switches per 100 person-years). The rate of switching remained steady at approximately 1.0% of all active patients in each calendar year since 2013 (Table 4).

**Table 4:** Trend of Second-Line Regimen Switching, by Year from 2010 to 2017 (*n* = 53,074)^a^

	2010	2011	2012	2013	2014	2015	2016	2017
Total # of active ART patients	3913	8943	16,003	24,099	31,077	35,610	32,363	29,077
# of switching patients	21	32	58	295	306	373	344	279
% of patients switched	0.54%	0.36%	0.36%	1.22%	0.98%	1.05%	1.06%	0.96%
# of switching patients with unsuppressed VL status	0	0	0	22	48	83	171	230
% of patients switching with unsuppressed VL status	0	0	0	7.46%	15.69%	22.25%	49.71%	82.44%

^a^ Among ART patients with at least 18 months of follow-up time (see Table 1 for characteristics of 53,074 ART patients). Year 2017 includes data from January –September (9 months) only. VL status refers to status prior to switching

**Fig. 4:**
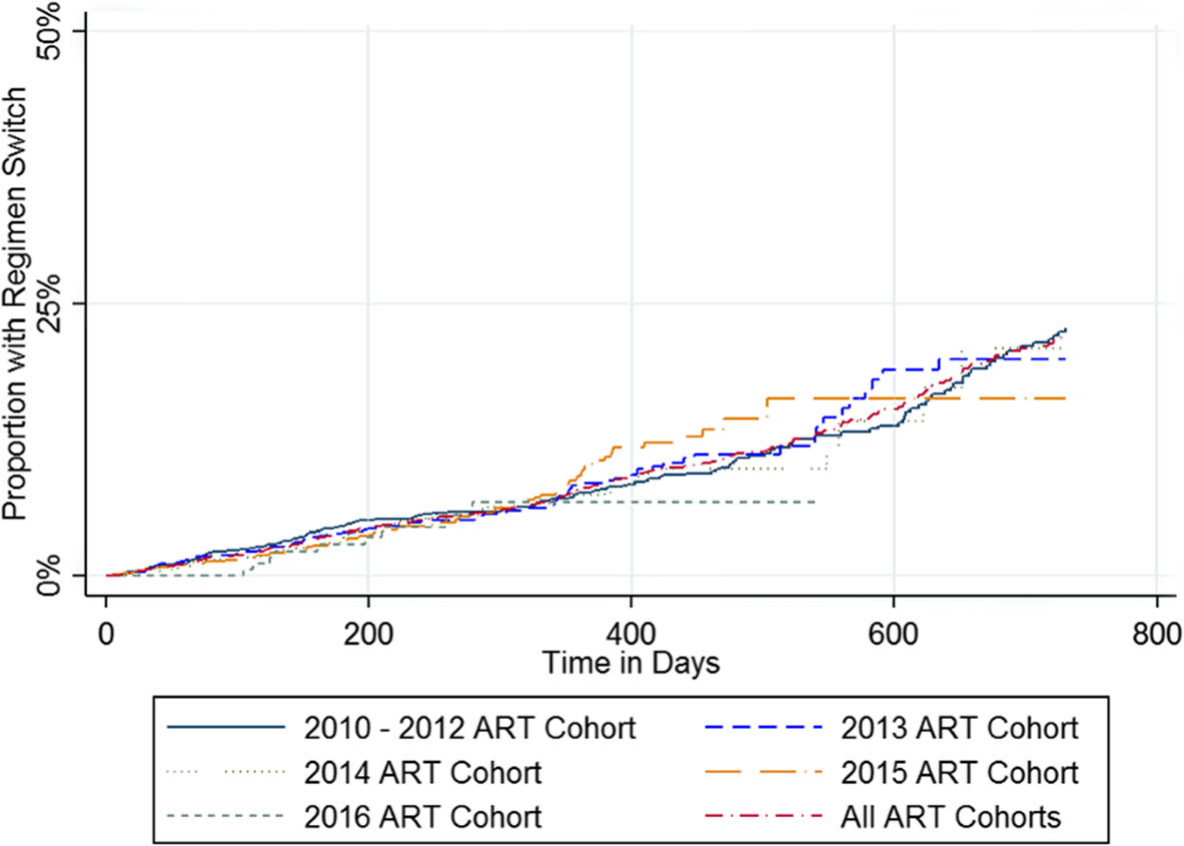
Kaplan-Meier Estimates of Time to Second-Line Regimen Switch Following Initial Unsuppressed Viral Load Test (*n* = 7468)

**Fig. 5:**
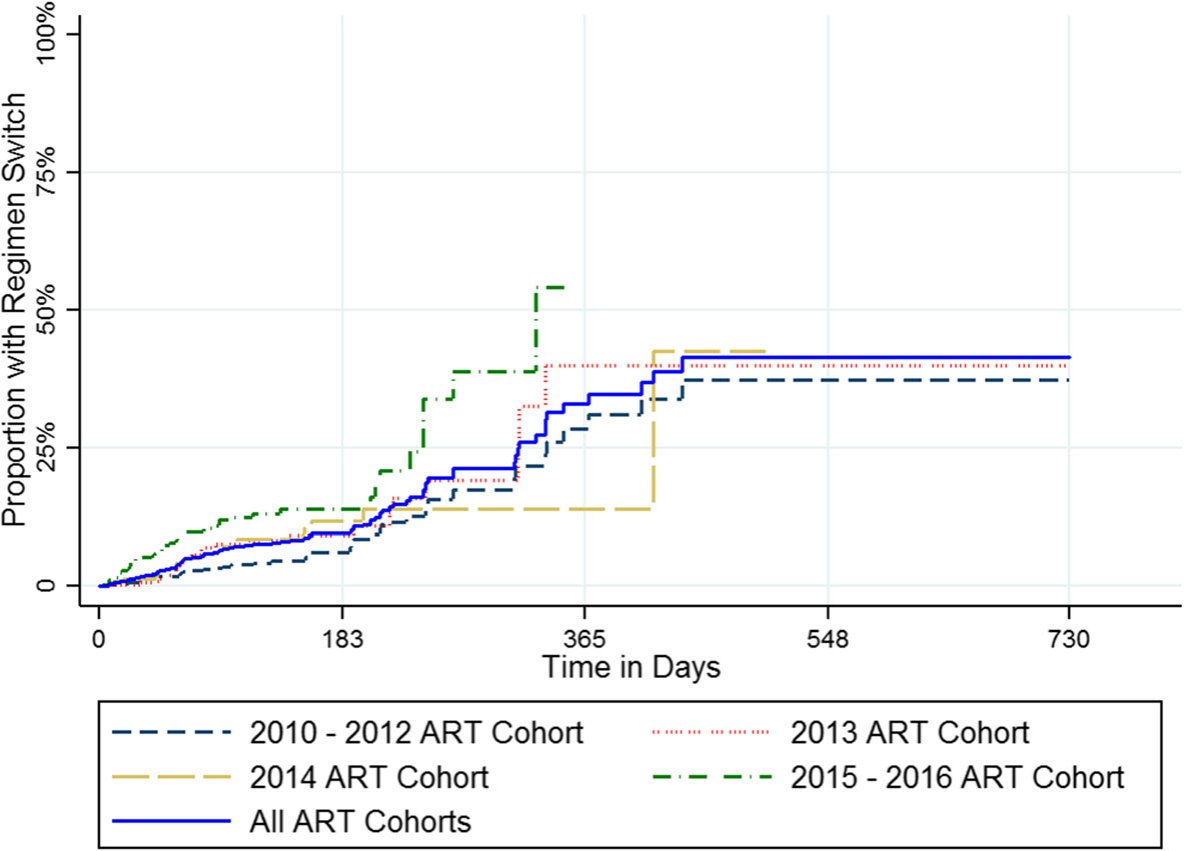
Kaplan-Meier Estimates of Time to Second-Line Regimen Switch Following Confirmed Viral Load Failure (*n* = 2102)

Figure 4 shows the timeliness of switching to second-line regimens after a first unsuppressed VL test result, based upon the Kaplan-Meier method. The overall proportion of patients who switched was estimated to be below 25% within 2 years of the initial unsuppressed VL. Timeliness of ART switching was similar across ART cohorts (Fig. 4). Figure 5 shows the timing of switching following confirmed VL failure, also based upon the Kaplan-Meier method. Among patients with confirmed VL failure, only 41.6% were estimated to switch to a second-line regimen within 2 years of confirmed VL failure (Fig. 5).

### Viral load status prior to switching to second-line ART

**Fig. 6:**
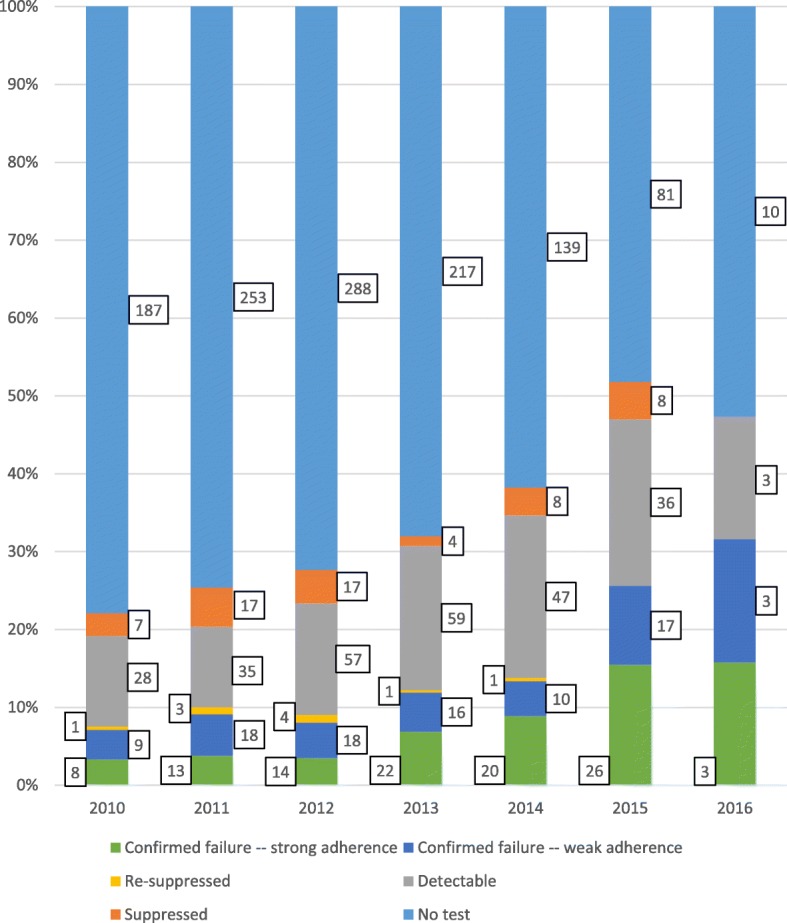
Viral Load Status Prior to Second-Line Regimen Switch, by ART Cohort (*n* = 1708) * *Analysis limited to second-line regimen switches among patients with at least 18 months of follow up

Among the 53,074 patients with at least 18 months of observed follow-up time, a total of 1708 patients switched to a second line-regimen (Fig. 6). The proportion of switching patients with a confirmed failure was higher among later ART cohorts. For the 2010 ART cohort, 240 were observed to switch to second-line ART, and only 17 (7.1%) had a confirmed failure before switching. For the 2015 ART cohort, a total of 168 patients were observed to switch, and 43 (25.6%) had a confirmed failure before switching. This increase in confirmed failures prior to switching included increases in cases both with and without evidence of strong adherence (Fig. 6).

### Association between viral load testing and second-line regimen switching

Our exploratory analysis of the association between VL testing and regimen switching showed that patients with confirmed VL failure had a 22.2 times higher hazard (95% CI for hazard ratio [HR]: 18.8–26.3; *p* < 0.001) of switching to a second-line regimen compared with those with no VL test done, after adjusting for age, gender, WHO stage at ART initiation, and year of ART initiation (Table 5). Those with a single unsuppressed VL result (without evidence of re-suppression) had a 5.6 times higher hazard (95% CI for HR: 4.9–6.5; *p* < 0.001) and those with a re-suppressed result had 2.3 times higher hazard (95% CI for HR: 1.3–4.4; *p* = 0.008) of switching compared with patients with no test. In contrast, patients who had suppressed VL result had a 0.4 times lower hazard (95% CI for HR: 0.3–0.5; *p* < 0.001) of switching compared to those with no test. Compared with patients at 16–24 age group when initiating ART, patients over age 55 had a 0.7 times lower hazard for switching to a second-line ART regimen (95% for HR: 0.6–0.9, *p* < 0.01), while comparisons with the 25–34, 35–44, 45–54 year age groups were not statistically significant. Males had a 1.4 times higher hazard for switching compared with females (95% for HR: 1.2–1.5, *p* < 0.001) in adjusted analyses. Patients with a more advanced WHO stage at ART initiation also had a higher hazard for switching to second-line ART in adjusted analyses. Compared with WHO stage I patients, those with stage II, III, and IV had 1.4 (95% for HR: 1.1–1.8, *p* < 0.01), 1.9 (95% for HR: 1.6–2.4, *p* < 0.001), and 3.2 (95% for HR: 2.6–3.9, *p* < 0.001) times higher hazard for switching to a second-line ART regimen, respectively (Table 5).

**Table 5:** Association between VL Testing and Second-Line Regimen Switching (*n* = 66,042)^a^

		N	HR	95% CI	*p*-value
VL test status	No test	36,442	1.0	Ref.	Ref.
	Suppressed	19,829	0.4	(0.3, 0.5)	< 0.001
	Unsuppressed	6022	5.6	(4.9, 6.5)	< 0.001
	Re-suppressed	1328	2.3	(1.3, 4.4)	< 0.01
	Confirmed failure	2421	22.2	(18.8, 26.3)	< 0.001
Age group	16~24	8173	1.0	Ref.	Ref.
	25~34	22,335	1.0	(0.9, 1.2)	0.89
	35~44	18,828	0.9	(0.8, 1.1)	0.45
	45~54	10,778	0.8	(0.7, 1.0)	0.09
	> 55	5928	0.7	(0.6, 0.9)	< 0.01
Gender	Female	42,623	1.0	Ref.	Ref.
	Male	23,419	1.4	(1.2, 1.5)	< 0.001
Baseline WHO stage	Stage 1	12,879	1.0	Ref.	Ref.
	Stage 2	10,983	1.4	(1.1, 1.8)	< 0.01
	Stage 3	14,217	1.9	(1.6, 2.4)	< 0.001
	Stage 4	21,814	3.2	(2.6, 3.9)	< 0.001
	Missing	6149	1.4	(1.1, 1.9)	0.01
Year of ART initiation	2010–2012	18,418	1.0	Ref.	Ref.
	2013	10,812	0.7	(0.6, 0.8)	< 0.001
	2014	11,629	0.6	(0.5, 0.7)	< 0.001
	2015	10,483	0.8	(0.6, 0.9)	< 0.01
	2016–2017	14,700	0.5	(0.4, 0.7)	< 0.001

^a^Includes all ART patients (see Table 1 for characteristics of 66,042 ART patients)

## Discussion

From 2010 to 2017, in conjunction with the growth of the national ART program and the expansion of criteria for ART eligibility, Haiti markedly increased the coverage of VL testing for ART patients. However, the coverage of VL testing must increase still further to meet national and international guidelines for HIV clinical management. Forty eight percent of active ART patients had no VL test done in 2017. Meanwhile, the percentage of patients switching to second-line regimens remained low, at around 1% annually. Among those patients with unsuppressed results, almost 60% of patients had no confirmatory testing as directed in WHO and Haitian national guidelines. Weak adherence to treatment is a considerable concern and seems to have been present in just over half of the cases of confirmed VL failure. The almost half of patients with good adherence but with confirmed failure is suggestive of the presence of ART drug resistance, [35] though this finding may be attenuated if the presence of “good adherence” was imprecisely or incompletely understood or documented.

Our findings add to the literature on the scale-up of VL testing in resource-limited settings. Jean-Louis et al. (2017) conducted research assessing VL outcome among patients receiving ART at five hospitals around Port-au-Prince, Haiti from 2013-15 [24]. Among 7903 patients on ART for 6 months or longer, 2313 patients (29.27%) received at least one VL test, a much higher proportion than the level of < 5% observed during 2013–15 in our national-level study. It is not surprising that Port-au-Prince, as the capital and the densest urban area of Haiti, would have gained greater access to routine VL testing at an earlier point in time compared with the rest of the country. Our results demonstrate that MSPP extended VL availability nationally after 2016, though at levels short of those needed to demonstrate achievement of the UNAIDS 95–95-95 targets for HIV epidemic control. This suggests the need for further understanding the gap in VL monitoring: whether these tests were not ordered, the results were not returned to the sites, or the results were not recorded in the EMR. Further progress in extending access to VL testing is needed to meet WHO and Haitian national clinical guidelines for ART monitoring.

In terms of virologic status as revealed by routine VL testing, our results were similar to those reported by Jean-Louis et al. (2017); both studies showed virologic suppression in around two-thirds of those tested in each ART cohort of patients. This low absolute level of suppression could indicate a preferential use of VL testing among those with clinical suspicion of treatment failure. Our finding that use of second-line regimens was limited to 1.4 switches per 100 person-years, is consistent or slightly lower than levels in other resource-limited settings [36]. In a cohort study involving patients from 16 sub-Saharan Africa countries from 2004 to 2013, a rate of 1.63 switches per 100 person-years was observed [25].

Before 2016, VL testing was not widely used as evidence for second-line switching in Haiti. But since 2016, VL testing has become a guide for clinicians to switch ART regimens. In terms of the association between VL testing status and second-line regimen switching, patients with confirmed VL failure were far more likely to be switched to second-line regimen compared with patients without a VL test. While this indicates that the VL test provides useful evidence to motivate health worker decisions to switch patients to second-line regimens, there remain gaps in timeliness of confirmatory testing and of regimen switching in Haiti. The delays in switching exceeded the timeframes described in the national ART guidelines for patient management. Fewer than half of patients who experienced confirmed VL failure switched to a second-line ART regimen within the 2 years following the confirmed failure. Such delays can increase the risk of developing high-level resistance to first-line ART, and of transmitting a resistant form of HIV to others.

In theory, the broader use of VL testing should result in optimized HIV clinical management, with timely detection of unsuppressed VL, timely confirmation of VL failure, and timely switching to a second-line ART regimen in cases where ART adherence is strong and resistance is suspected. Our study found a mixed pattern where lack of confirmatory testing after unsuppressed VL results persisted through time, and timeliness of switching to second-line regimens also failed to improve markedly in recent ART cohorts. Confirmatory testing is necessary to determine effectiveness of enhanced adherence counseling and to inform clinical decisions about treatment options. Our findings suggest that use of confirmatory testing for VL failure and use of second-line regimens do not align with national guidelines. Even while expanding coverage of initial routine VL tests for ART monitoring, it is also important to prioritize repeat VL testing among those with unsuppressed results. Simply providing VL testing without being prepared to manage those with unsuppressed results is problematic. Indeed, Haiti has had underused stocks of second-line regimens available at the national level in the past [37]. It is necessary for Haiti to continue expanding access to VL testing, and to ensure timely return of VL results so that clinicians can make decisions about enhanced adherence counseling and switching. Expanded evidence-based initiatives to promote strong ART adherence are needed, as are initiatives to modify HIV clinical management practices, and to support timely switching for patients with confirmed virologic failure despite strong ART adherence.

### Limitations and strengths

This study only included VL tests with results reported within the iSanté EMR system; some tests may have been done but without results returned to iSanté (reflecting a backlog of tests and results in Haiti’s reference laboratories). Our results cannot be taken as representative for all ART clinics in Haiti, since they exclude certain ART clinics from the GHESKIO and Partners in Health networks which did not use iSanté as well as 35 ART clinics using the iSanté data system but without current, reliable data saved to the central iSanté data repository. Exclusion of iSanté sites without reliable data may have biased our results. Weaknesses in routine clinical information systems may be associated with other health systems weaknesses (e.g. laboratory systems, supply chain systems), but we cannot know if bias extended to results related to HIV viral suppression.

Other methods of monitoring effectiveness of ART using clinical or immunologic markers (such as CD4 monitoring, which was the primary approach before 2015) were not considered in this study. Also, our study was not able to track patients who transferred care between health facilities or to detect cases where the same person may have been registered in the iSanté EMR using more than one patient identifier, meaning that our patient counts may have included duplicate records for the same person. Prior studies have estimated that the level of duplicate records within iSanté is approximately 8–10% [38, 39]. Our results on the proportion of patients with unsuppressed VL tests should not be interpreted as a population-level estimate, since a high proportion of patients had no VL test and clinicians may have preferentially ordered VL tests for patients with clinical or immunologic signs of ART treatment failure.

This study is the first to describe the routine uptake of VL test and its relationship to second-line ART regimen switching on a national scale in Haiti. The study included 66,042 patients from 88 sites from all regions of the country, accounting for around half of Haiti’s HIV patients over an almost 8 year period, meaning the results are meaningful in presenting a picture of national scale-up of VL testing and regimen switching. Based on these strengths, our study can inform policymakers and related stakeholders on key gaps, particularly the low rate of repeat VL testing after unsuppressed results and low rates of regimen switching, especially among women and among those who started ART at an early asympto-matic WHO stage. These gaps are important to address in order to reach the 95–95-95 goals for HIV epidemic control.

## Conclusions

Our study shows marked expansion of access to VL testing, especially since 2016, though still with half of patients not receiving an annual VL test. Clearly, VL monitoring will need to be increased to ensure achievement of 95–95-95 targets. Our study suggests use of second-line regimens changed little with time, and the lag in switching patients to second-line ART regimens remains a concern. Greater access to VL testing seems to have supported clinical decision-making about ART switching to some degree, but with progress still needed in order to achieve appropriate use of second-line regimens in Haiti. In the context of broadening access to VL testing overall, use of timely confirmatory VL testing is needed in order to guarantee that patients are placed on appropriate medication in cases of likely HIV drug resistance. To guarantee that HIV patients obtain effective treatment and avoid development of drug resistance, healthcare workers must be supported to ensure confirmatory testing and to take rapid action after confirmed VL failure, so that Haiti’s national ART monitoring guidelines move from theory to practice.

## Data Availability

The data that support the findings of this study are available from the Haiti Ministère de Santé Publique et de la Population (MSPP) but restrictions apply to the availability of these data, which were used under a data use agreement for the current study, and so are not publicly available. Data are available from the authors upon reasonable request and with permission of the Haiti Ministère de Santé Publique et de la Population (MSPP).

## Abbreviations

ART: Antiretroviral Therapy
ARV: Antiretroviral
CDC: Centers for Disease Control and Prevention
CI: Confidence Interval
EMR: Electronic Medical Record
HR: Hazard Ratio
IQR: Interquartile range
MPR: Medication possession ratio
MSPP: Ministry of Public Health and Population
NNRTI: Non-nucleoside reverse transcriptase inhibitor
NRTI: Nucleoside reverse transcriptase inhibitor
PEPFAR: President’s Emergency Plan for AIDS Relief
VL: Viral Load
WHO: World Health Organization
